# Phylogenetic estimation error can decrease the accuracy of species delimitation: a Bayesian implementation of the general mixed Yule-coalescent model

**DOI:** 10.1186/1471-2148-12-196

**Published:** 2012-10-02

**Authors:** Noah M Reid, Bryan C Carstens

**Affiliations:** 1 Department of Biological Science, Louisiana State University, Baton Rouge, LA, 70803, USA; 2 Department of Ecology, Evolution and Organismal Biology, The Ohio State University, Columbus, OH, 43210, USA

**Keywords:** Species delimitation, GMYC, Bayesian phylogenetics, DNA barcoding

## Abstract

**Background:**

Species are considered the fundamental unit in many ecological and evolutionary analyses, yet accurate, complete, accessible taxonomic frameworks with which to identify them are often unavailable to researchers. In such cases DNA sequence-based species delimitation has been proposed as a means of estimating species boundaries for further analysis. Several methods have been proposed to accomplish this. Here we present a Bayesian implementation of an evolutionary model-based method, the general mixed Yule-coalescent model (GMYC). Our implementation integrates over the parameters of the model and uncertainty in phylogenetic relationships using the output of widely available phylogenetic models and Markov-Chain Monte Carlo (MCMC) simulation in order to produce marginal probabilities of species identities.

**Results:**

We conducted simulations testing the effects of species evolutionary history, levels of intraspecific sampling and number of nucleotides sequenced. We also re-analyze the dataset used to introduce the original GMYC model. We found that the model results are improved with addition of DNA sequence and increased sampling, although these improvements have limits. The most important factor in the success of the model is the underlying phylogenetic history of the species under consideration. Recent and rapid divergences result in higher amounts of uncertainty in the model and eventually cause the model to fail to accurately assess uncertainty in species limits.

**Conclusion:**

Our results suggest that the GMYC model can be useful under a wide variety of circumstances, particularly in cases where divergences are deeper, or taxon sampling is incomplete, as in many studies of ecological communities, but that, in accordance with expectations from coalescent theory, rapid, recent radiations may yield inaccurate results. Our implementation differs from existing ones in two ways: it allows for the accounting for important sources of uncertainty in the model (phylogenetic and in parameters specific to the model) and in the specification of informative prior distributions that can increase the precision of the model. We have incorporated this model into a user-friendly R package available on the authors’ websites.

## Background

A common challenge faced by empirical researchers in studies of ecological communities is to identify individuals at the species level from limited information collected from a broad taxonomic range of organisms. In many cases, useful taxonomic keys for particular groups or regions are not available. This is because many diverse groups are morphologically cryptic, contain many undescribed taxa, or existing taxonomic literature is conflicting, an issue referred to as the “taxonomic impediment” [1]. In these cases, short DNA sequence tags (the DNA barcode region of the mitochondrial gene COI, or a hypervariable region of the microbial 16S rRNA gene) are frequently surveyed because they can be rapidly and inexpensively collected [2,3]. DNA barcoding initiatives aim to connect these sequence tags to taxa validated by expert taxonomists [4,5], but at present this is not possible for most groups. As a result, diversity must frequently be quantified in the absence of a low-level taxonomic framework. In order to accomplish this, observed DNA sequences must be clustered into putative species. While the delimitation of species is a complex philosophical and biological problem [6], species concepts widely share the idea that species are independently evolving metapopulation lineages [7]. This provides a justification for using genetic data (such as DNA barcodes) as the primary data for the diagnosis of these lineages, as they contain the signal of historical processes involved in lineage divergence [8]. As a caveat, lineages identified in this way will not necessarily meet the criteria for species status under any given species concept, such as reproductive isolation from other such lineages, or exhibit morphological, ecological or behavior divergence.

Methods used for delimitation of species from barcode data are a subset of those developed for the larger problem of species delimitation. They can be considered species discovery methods because they must be functional in the absence of good *a priori* taxonomic information [9-11]. This contrasts with validation methods (e.g. [9,12]), which test specific hypotheses of species status, and assignment methods, which assign unknown individuals to existing species (e.g. [13-16]). Of the handful of approaches typically used to discover species limits using genetic data, thresholds based on pairwise sequence distances among individuals are perhaps most commonly applied to cluster sequences into putative species [5,17]. These methods identify some level of sequence divergence beyond which two individuals cannot be considered the same species. Distance threshold methods have been criticized because they do not account for evolutionary processes [18], and the uncertainty in selecting an appropriate threshold [15], which relies on an observable “barcode gap” between pairwise intraspecific and interspecific DNA sequence distances ( [19-22]; but see [23]).

Pons et al. [24] introduced a model-based alternative to distance threshold methods. The model, the general mixed Yule-coalescent (GMYC), takes a phylogenetic tree estimated from DNA sequence data and assumes that the branching points in the tree correspond to one of two events: divergence events between species level taxa (modeled by a Yule process [25]), or coalescent events between lineages sampled from within species (modeled by the coalescent [26]). Because the rate of coalescence within species is expected to be dramatically greater than the rate of cladogenesis, the GMYC aims to find the demarcation between these types of branching. This model has been shown to be useful in several empirical studies [24,27-31]. Because it is based on a Likelihood function that directly models evolutionary processes of interest, it provides a means to ameliorate some of the criticisms leveled at threshold methods. Notably, it has allowed for quantification of uncertainty in delimitation of species [32] and avoids the use of non-independent pairwise sequence distances (e.g. in [23]) as data.

The GMYC model as presently implemented, however, does not account for three potentially large sources of error. First, it is widely recognized that a variety of factors can cause the genealogy from a particular locus to be discordant with the true history of speciation [33], and the GMYC, like all methods based on a single locus, can be mislead by this discordance. Second, there may be error in the model estimates. Under certain circumstances, the transition from speciation events to coalescent events may be indistinct (e.g., a combination of rapid speciation events and large effective population sizes) causing the model to have a wide confidence interval. A recent implementation by Powell [32] accounts for uncertainty in the threshold parameter and produces model-averaged species limits, but uses point estimates for the other parameters. Finally, phylogenetic error can diminish the accuracy of delimitation results. The GMYC model requires the user to input a point estimate of the phylogenetic tree and inference is premised on the accuracy of this point estimate. Diversity studies using sequence tags, however, typically use relatively short loci that yield estimates of topology and branch lengths that may have high levels of uncertainty. This uncertainty could influence the accuracy of the model.

In order to address the second and third potential sources of error, we introduce a Bayesian implementation of this model with flexible prior distributions in the statistical scripting language R [34]. It accounts for the error in phylogenetic estimation and uncertainty in model parameters by integrating over uncertainty in tree topology and branch lengths and in the parameters of the model via Markov Chain Monte Carlo simulation (MCMC) [35]. It produces marginal posterior probabilities for species that are independent of these factors along with output characterizing the posterior distribution that is suitable for downstream analyses of community structure accounting for uncertainty in species limits and phylogeny using R packages such as Picante [36], Vegan [37], and APE [38]. We also conduct simulation tests to evaluate the performance of the model and re-analyze a dataset previously analyzed with the Likelihood version of the model.

## Methods

### Model

Given an ultrametric phylogenetic tree estimated from a set of sequences consisting of multiple species and multiple individuals within species, the GMYC model decomposes the tree into its component waiting times between branching events. These waiting times are the data to be modeled [21]. The parameter of primary interest is a threshold parameter before which the waiting times are modeled according to a Yule process and after which, according to *k* intraspecific coalescent processes, where *k* is the number of species with more than one unique sequence sampled. The Likelihood of a waiting time under a Yule model is:

$$L_{\left(x_{i}\right)}=\lambda n_{i}^{p}e^{-\lambda n_{i}^{p}x_{i}}$$

where the waiting times (*x*_*i*_) are assumed to be exponentially distributed and a function of: the branching rate (*λ*), the number of lineages in the interval (*n*_*i*_), and a rate change parameter that accounts for the possibility of increasing or decreasing diversification rate with time (*p*; Pons et al. [24]). The Likelihood (*L*) of a waiting time under the coalescent model is:

$$L_{\left(x_{i}\right)}=\lambda n_{i}{\left(n_{i}-1\right)}^{p}e^{-\lambda n_{i}{\left(n_{i}-1\right)}^{p}x_{i}}$$

where the branching rate (λ) can be interpreted as 1/N_e_μ (where μ is the per generation mutation rate, or the number of generations per year, depending on the branch length units of the tree) and the rate change parameter (*p*) can be interpreted as population size change over time [24].

The GMYC model combines the above models, and the Likelihood of the full model is calculated by assigning lineages in each waiting interval to either the Yule process or one of the coalescent processes such that:

$$b=\lambda_{k+1}n_{i_{,k+1}}^{p_{k+1}}+\sum_{j=l,k}\lambda j{\left(n_{\mathrm{ij}}\left(n_{\mathrm{ij}}-1\right)\right)}^{p_{j}}$$

Making the full Likelihood of a waiting time:

$$L_{x_{i}}=be^{-bx_{i}}$$

where *k* indexes the branching processes (1:*k* are intraspecific coalescent processes, *k + 1* is the Yule process), *λ*_*k+1*_ and *p*_*k+1*_ are the branching rate and rate change parameters for the Yule process, and *λ*_*j*_ and *p*_*j*_ are the branching rate and population size change parameters for the coalescent process. Following Pons et al. [24], we constrain *λ*_*j*_ and *p*_*j*_ to be identical across coalescent processes. The number of lineages assigned to the Yule and coalescent processes in each waiting interval are *n*_*i,k+1*_ and *n*_*i,j*_, respectively. Assignment of lineages in this case is determined by the selection of a threshold.

Because the sequence data employed in these analyses are typically from short fragments, and thus likely to yield trees with high levels of uncertainty in topology and branch lengths we implemented this model in a Bayesian statistical framework. It eliminates the reliance on point estimates of the phylogeny and model parameters and by estimating the marginal probabilities of the identity of species, allows one to incorporate that uncertainty in downstream analyses. Our implementation operates as follows. First, the posterior distribution of trees and branch lengths are characterized using BEAST [39]. Second, a post-burn-in sample of the trees from that posterior distribution is taken, and for each tree, an MCMC simulation is conducted to characterize the posterior distribution of the GMYC model. Following Pons et al., we did not allow the *λ* parameters to be freely estimated, but used a Maximum Likelihood estimator [40]. The non-normalized posterior density function is as follows:

$$P\left(T,\lambda_{j},\lambda_{k+1},p_{j}p_{k+1}|\tau \right)\propto P\left(T,\lambda_{j},\lambda_{k+1},p_{j}p_{k+1}\right)P\left(\tau |T,\lambda_{j}\lambda_{k+1},p_{j}p_{k+1}\right)$$

where *T* is the threshold. Because each MCMC evaluates the posterior of the GMYC conditioned on the tree, pooled samples from analyses of many trees sampled from the tree posterior result in a posterior probability distribution of species delimitations conditioned only on the sequence data, the phylogenetic model and the GMYC model.

### Simulation testing

We evaluated the utility of this implementation of the GMYC using three simulation experiments. In each, we simulated gene trees from species trees using ms [41]. All species trees contained 50 species and were generated via a Yule process in Mesquite [42], randomly sampling 50 species from a tree of 150 species. This design was intended to mimic environmental samples of a given taxon, which would not be expected to contain all species in a clade.

In the first experiment we examined the effect of tree depth on model accuracy. We simulated 50 species trees as above and scaled them to four different depths (20 N, 40 N, 80 N, 160 N generations, where N is the effective number of diploid individuals in the species). When considering how the results translate to haploid, maternally inherited organellar DNA, the equivalent tree depths are halved (e.g. 10 N, 20 N…) and N becomes the effective number of females in the population. We then simulated a single gene tree from each species tree at each depth, sampling five alleles per species. For each of these trees we sampled from the posterior for 100,000 generations, discarding the first 10,000 generations as burn-in and thinning every 100 generations, assessed stationarity by examining plots of the parameters by eye, and characterized the posterior distribution of the threshold parameter, which determines the species limits given a tree. Priors on all parameters were uniform distributions; in the case of the threshold parameter, from U(2,250) and for the *p* parameters U(0,2).

In the second experiment we looked at the influence of sampling. The species trees with a depth of 80 N from the first experiment were used with four different sampling schemes: 2 alleles per species, 5 alleles per species, 10 alleles per species, and a random number of alleles per species, drawn from a lognormal distribution, with a mean and standard deviation of 1 (an average of 5 alleles per species; approximately 17% of species were represented by singletons). We used the lognormal distribution because it approximates some real species-abundance distributions [43] that might be observed in actual species delimitation datasets. We conducted the analyses as in the first experiment.

In the third experiment, we tested the effect of nucleotide sampling and tree estimation on the accuracy of the model (in our simulations, sequence length is directly correlated with the number of variable sites). We selected 10 of the simulated gene trees from 10 species trees scaled to 160 N generations for which the confidence intervals in the analysis overlapped the true value of 50 species. We then simulated DNA sequences on those gene trees of 300 bp, 600 bp, 1200 bp and 2400 bp using Seq-Gen [44]. We assumed *θ* = 0.015 (corresponding to an N_e_ of 250,000 and a mutation rate of 1.5% per million generations) and an HKY + G model. We characterized the posterior distribution of trees using the true model of sequence evolution and a strict clock model in BEAST. We pruned all identical sequences and ran BEAST for 10 million generations, discarding the first million as burn-in, at which point all parameters for all replicates had effective sample sizes above 150 and most above 200. We then ran independent GMYC MCMC analyses on 100 trees sampled every 50,000 generations from the BEAST posterior distribution of trees, pooled the results and characterized the marginal posterior distribution of the threshold parameter compared to the distribution produced using the true tree.

### Empirical data analyses

To illustrate how this implementation of the GMYC could be applied; we downloaded from GenBank and reanalyzed the dataset from Pons et al., the original publication of the GMYC (Coleoptera:Carabidae:*Rivacindela*; AJ617921–AJ618351, AJ618352–AJ618766, AJ619087–AJ619548; [24]). We first pruned each alignment to consist only of unique sequences. Since we are not using a true genealogy sampler (*sensu*[45]), identical sequences will result in many zero-length branches at the tip of the tree, and will cause the model to over-partition the dataset. We then applied a phylogenetic clock model to estimate the posterior distribution of ultrametic trees using BEAST. We partitioned models of sequence evolution by codon, and also by gene when multiple genes were used, applying models of sequence evolution selected by DT ModSel [46]. We accepted that runs converged when all parameters reached ESS values greater than 200 and checked that posterior distributions differed from priors. We explored the use of different tree priors as it is conceivable that in cases where branch-length information is lacking, the prior could strongly influence the posterior. For Bayesian GMYC MCMC analyses, we ran each tree for 10,000 generations, discarding the first 1000 as burn-in and sampling every 100 generations. Using 100 trees sampled from the BEAST posterior distribution of trees, this resulted in 9000 samples. We selected this length of Markov-chain because preliminary analyses suggested that stationarity was usually achieved by 1000 generations. We compared the posterior distribution from sampling multiple trees to that from the maximum clade credibility tree and examined the effect of changing the prior on the Yule rate change parameter (*p*_*k+1*_*).* We compared the posterior distribution to the point estimate produced by the Likelihood version of the model, and to the Akaike weights [47] of each threshold point.

## Results and Discussion

### Simulation tests

We first tested the influence of tree depth on model performance. When deeper trees are simulated, coalescent and Yule branching processes are expected to occur on more distinct time scales, and thus in general the model should perform better. The influence of tree depth is actually confounded by two issues, however. First, as the tree depth becomes shallower the implied rate of speciation increases because all trees contain 50 species. If the rate of speciation approaches the rate of coalescence within species, then a sharp transition between processes should not be detectable. Second, as the implied rate of speciation increases, more species originate relatively recently. The expected time to coalescence for a diploid, panmictic population is 4 N generations. Cladogenic events occurring more recently than this are expected to be increasingly difficult to delimit for two reasons: they are more likely to yield species that are not monophyletic and thus impossible to accurately identify under this model, and the most recent common ancestor (MRCA) of the daughter species is more likely to occur more recently than the threshold point. Assuming species monophyly, the expected time to the MRCA for two species that diverged 4 N generations ago is 6 N generations. Therefore all probability should be on thresholds older than 4 N generations, and most on thresholds older than 6 N generations. Again, when considering maternally inherited, haploid, organellar DNA, equivalent times in N generations are halved, and N becomes the effective number of females in the population. This would give an expected time to MRCA of 3 N generations.

**Figure 1:**
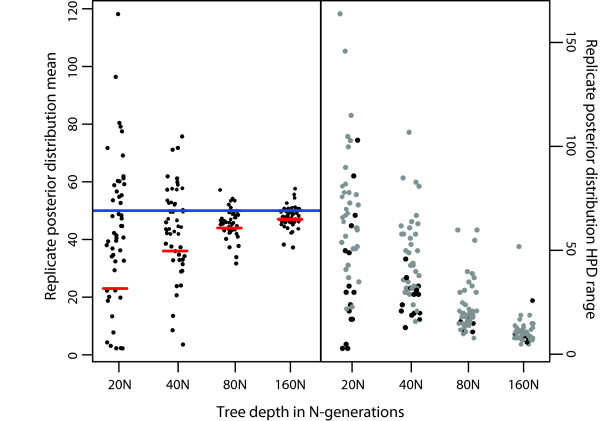
**Testing the effects of tree depth.** Legend: The left pane shows the effect of increasing tree depth on the threshold parameter (number of species). The dots are posterior means of individual replicates. The blue line shows the expected number of species (50) and the red line shows the mean number of species diverging earlier than 4 N generations. The right pane shows the 95% HPD interval for each replicate. Gray points indicate the HPD overlapped the true number of species, black ones that it did not.

The results of this test are dramatic (Figure 1, Additional file 1: Figure S 1). There is a clear increase in accuracy as well as a decrease in the range of the 95% highest posterior density interval (HPD) with increasing tree depth. At shorter tree depths, the model’s performance diverged from expectations. When trees are short, larger numbers of species have divergence times younger than 4 N generations and thus should not be detectable under the model. Therefore we expected the number of species delimited to be smaller than for deeper trees. We did not observe this. Instead, replicate posterior means became widely scattered around the true value of 50 species. 95% HPDs also did not uniformly increase with decreasing tree depth, instead, a wide distribution of HPDs were observed. We also expected threshold times to have a minimum value of approximately 4 N generations. At deeper tree depths this is observed, with 0.13% and 1.6% of MCMC steps sampling thresholds younger than 4 N generations for tree depths of 160 N and 80 N, respectively. However, at 40 N and 20 N tree depths, 20% and 40% of MCMC steps sampled thresholds younger than 4 N generations.

These results indicate that the model performs well under demographic or sampling conditions that result in coalescent and Yule processes occurring on very different time scales. It does not, however, perform optimally when those conditions are not met.

Ideally one would hope that as inference of the threshold point became more difficult, that the 95% HPDs would increase, but still encompass the true value 95% of the time. This is not the case at the 20 N and 40 N tree depths. HPDs generally become broader, but for increasing numbers of simulation replicates, they fail to encompass the true value. 50 species arising in 40 N generations constitutes a very rapid radiation, with an average of 89% of branches in the species tree shorter than the expected population coalescence time of 4 N generations. Failure to accurately assess credibility intervals in this case is likely because in this area of parameter space, the GMYC is no longer an accurate approximation of the real branching process in the gene tree. Rather than there being a threshold between coalescent and speciation branching processes, the two processes are intermixed because there is little time for the independent evolution of lineages prior to speciation. Note that these conditions will cause any DNA barcode-based method of species discovery to fail and will also challenge more realistic models utilizing multilocus data and prior information on population assignment.

**Figure 2:**
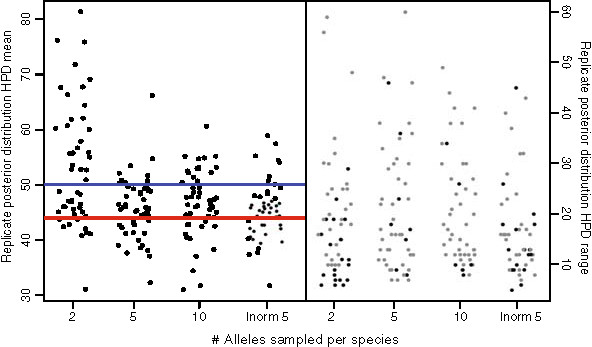
**Testing the effect of allele sampling within species.** Legend: We chose four sampling schemes: 2, 5, 10 alleles and a lognormally distributed (mean = 5) number of alleles per species. All species trees had a depth of 80 N generations. The left pane shows the effect of sampling scheme on the threshold parameter (number of species). The dots are posterior means of individual replicates. The blue line indicates the number of expected species (50) and the red line shows the mean number of species diverging earlier than 4 N generations. The right pane shows the 95% HPD interval for each replicate. Gray points indicate the HPD overlapped the true number of species, black ones that it did not.

Next we examined the effect of intraspecific sampling. Because the data points used by the model are waiting times between branching events, we expected that with 50 species, we would not need extremely high sampling to accurately characterize the model, and that the distribution of samples among species would not be particularly important. Our expectations were met. We found that sampling of 2 individuals per species yielded poor results (Figure 2, Additional file 1: Figure S 2). Replicate posterior means showed a strong bias towards inference of a large number of species. Sampling of greater than 2 individuals per species provided an improvement in the accuracy of the posterior means, but no change in the 95% HPD range. All sampling schemes greater than 2 individuals per species appeared to yield similar results, including the more realistic condition of a lognormal distribution of alleles among species in which a large number (~17%) of species are represented by singletons. While delimiting rare species, particularly from single specimens, is a challenge faced by taxonomists [48], our results suggest that the GMYC method may efficiently delimit species represented by singletons by calibrating the divergence threshold using data from better represented species.

**Figure 3:**
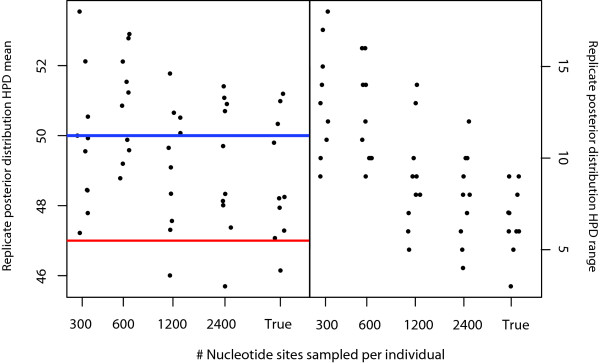
**Testing the effect of DNA sequence length.** Legend: We compared results from 300 bp, 600 bp, 1200 bp and 2400 bp with those estimated from the true tree. The left pane shows the effect of nucleotide sampling on the threshold parameter (number of species). The dots are posterior means of individual replicates. The blue line indicates the number of expected species (50) and the red line shows the mean number of species diverging earlier than 4 N generations. The right pane shows the 95% HPD interval for each replicate. Gray points indicate the HPD overlapped the true number of species, black ones that it did not.

Finally, we tested the effects of nucleotide sampling and the incorporation of phylogenetic uncertainty. We expected to find wider HPDs with less sequence data, as uncertainty, particularly in branch lengths should be greater. We found a mild reduction in accuracy of the posterior means with up to 600 bp of sequence, but after that, posterior means converged on those of the true tree. The 95% HPDs improved with the addition of more sequence, but had not quite converged on those estimated from the true tree, even at 2400 bp (Figure 3, Additional file 1: Figure S 3). This suggests that uncertainty in phylogenetic estimation, particularly in typical DNA barcode datasets of ~650 bp will contribute substantially to uncertainty in species delimitation.

Three factors that could influence the accuracy of the model that were not explored here: migration, population substructure and selection. Papadopoulou et al. [49] examined the effects of migration on the formation of detectable GMYC clusters. They simulated datasets under an island model and found that even very low levels of migration (far less than the *Nm* = 1 typically invoked as the limit for neutral population divergence) caused likelihood ratio tests to fail to reject the null model of a single branching process. They interpreted this as evidence that the likelihood implementation of the model is conservative and will not infer species at all unless they are strongly isolated.

Papadopoulou et al.’s simulations assumed complete demic sampling, but Lohse [50] conducted simulations showing that under moderate migration rates (*Nm* = 0.07) and with a large proportion (95%) of demes unsampled that spurious, significant clusters could be inferred from the true gene genealogies. In his simulations, Lohse showed that when 10 demes were sampled from a metapopulation consisting of 200 demes, that an average of 13 species were inferred, and 80% of replicates rejected the null model. Wakeley [51] described the genealogical pattern resulting from such a process as having two phases that occur on very different time scales: a scattering phase, in which there is rapid coalescence and migration in local demes, and a collecting phase that begins when each remaining lineage is in its own deme and takes a very long time. In this case the GMYC might see the scattering phase as the “coalescent process” and the collecting phase as the “Yule” process. Further exploration of this issue is likely to be important, particularly if the GMYC is applied to phylogenetic samples with deep phylogeographic sampling.

While Lohse shows convincingly that this interaction of parameter space with sampling can mislead the GMYC, it is not clear to what extent these problematic areas of parameter space exist in real datasets. We simulated 10 genealogies using ms under the conditions above and observed that the average time to coalescence of all lineages was 3,940 N generations (N is the size of a population in one deme), with the scattering phase taking the first 4-6 N generations. If we assumed that these 200 demes were species level taxa, each with *θ* = 0.01, we would expect to observe GMYC clusters with MRCAs at a depth of 0.01-0.015 substitutions per site and the MRCA for all lineages at 9.85 substitutions per site. It is unlikely that the collecting phase would have the time to play out under this scenario, as it would take nearly 500 million years at a mutation rate of 0.02 substitutions per site per million years. If, by contrast, we assume that these demes represent populations at a smaller scale, each with a theta of 0.01/200, then we would expect MRCAs of delimited clusters to be at a depth of 0.00005-0.000075 substitutions per site. With a typical DNA barcode or short mitochondrial DNA set of 650-2000 bp, the scattering phase would be undetectable. The MRCA for all lineages would occur at 0.049 substitutions per site. Unless this process was considered in the context of a larger species tree, it is unlikely that the GMYC would identify a significant branching threshold.

### Empirical analyses

**Figure 4:**
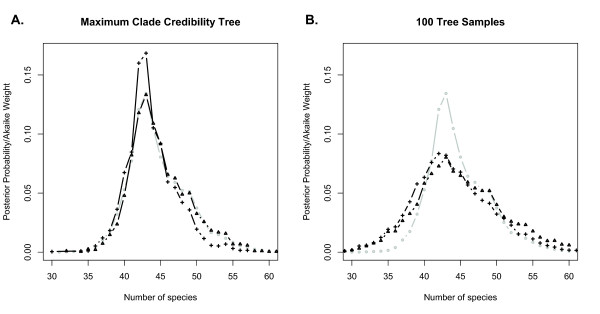
**Comparing Methodological Approaches.** Legend: We compared the new Bayesian implementation with the Likelihood method, with the effect of varying priors, and the inclusion of phylogenetic uncertainty. 4**A** shows Akaike Weights from the Likelihood method at each threshold point (gray circles), posterior probabilities given a U(0,2) prior (black triangles) and a U(0,1,2) prior (black crosses) on the Yule rate-change parameter. All three results were calculated from the maximum clade credibility tree. 4**B** shows the same results, except the posterior probabilities were calculated by running the analysis on 100 trees sampled from the posterior distribution of trees generated in BEAST.

**Figure 5:**
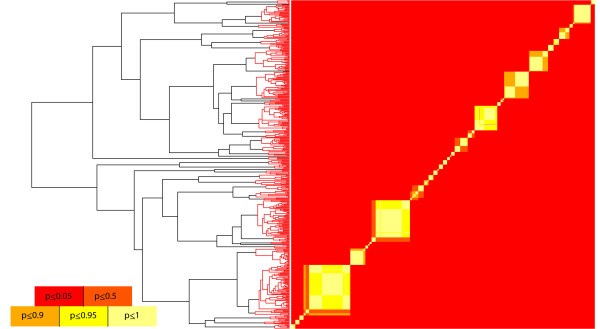
**Summary of empirical analyses.** Legend: We compare the results of Likelihood and Bayesian analyses of the Pons et al. *Rivacindela* dataset. The phylogenetic tree is the maximum clade credibility tree from BEAST and the clades highlighted in red represent the Maximum Likelihood species limits. The colored table is a sequence-by-sequence matrix. Cells are colored by the posterior probability that the corresponding sequences are conspecific, and this allows for the visualization of uncertainty in species limits. Off-diagonal color patterns indicate that some uncertainty in species limits owes to uncertainty in the topology of the phylogeny.

We reanalyzed the empirical data used by Pons et al. to illustrate the original formulation of the GMYC so as to provide a direct comparison of the implementations using representative data. The BEAST run converged after 27 million generations and we discarded 2.7 million trees as burn-in. The estimate of the standard deviation of the lognormal distribution of rates did not overlap 0, so we could not use a strict clock with these data. When using samples of trees from the BEAST posterior distribution, the mean number of species estimated by the Bayesian GMYC was 44 and the 95% HPD ranged from 34 to 57. The rate change parameter for the Yule process ranged as high as 1.9. In this model, the fold change in speciation rate from the root to the last speciation event is equal to *n*^*p*^/*n* where *n* is the estimated number of species and *p* is the Yule rate change parameter. In this case, given 44 species, *p* = 1.9 allows for a 30-fold speciation rate increase. We thought this might be unrealistically high (A sampling of three recent papers examining diversification rate shifts yielded a maximum increase from the background rate of approximately 8-fold [52-54]), and thus re-ran the analysis with the prior distribution set to U(0,1.2), or a maximum 2-fold increase. This minimally influenced the results. We also analyzed the maximum clade credibility tree under both priors, and using the likelihood implementation of the GMYC. We compared the results of Likelihood and Bayesian analyses by calculating Akaike weights for each possible threshold in the Likelihood analysis. Akaike weights are the relative likelihoods of a set of models and thus suited for qualitatively comparing results among these analyses. The Bayesian GMYC analysis of the maximum clade credibility tree yielded a mean of 44 species and a narrower 95% HPD of 38–55 species. When applied to the maximum clade credibility tree, the U(0,1.2) prior did change the results, yielding a posterior mean of 43 species and a 95% HPD of 38 to 51. The Maximum Likelihood analysis resulted in 44 species with a 95% confidence interval of 39 to 51 species. The results of the Likelihood and Bayesian analyses of the maximum clade credibility tree are very similar (Figure 4a), particularly when the prior U(0,2) is placed on the Yule parameter, but when sampling trees, the posterior distribution is substantially broader (Figure 4b). At least some of this uncertainty stems from variation in topology. Plots of pairwise probabilities of conspecificity demonstrate this (via off-diagonal variation in probability when ordered by a single tree; Figure 5). The probability distribution of the number of species in the sample is also wider for the posterior distribution than for the Akaike weights ( [47], Figure 4). Our Bayesian approach is similar in spirit to the model-averaging approach of Powell [32] in that its goal is to make the inference of species limits and analyses based on them more robust to uncertainty. There are, however, three major differences. First, we take into account phylogenetic uncertainty. As indicated by our results, this is perhaps the most influential difference, although in theory it could also be accounted for using model averaging. Second, our Bayesian method requires the specification of prior knowledge. Depending on a researcher’s comfort with assigning prior probability distributions to the rate and threshold parameters, this is either an advantage or a disadvantage. Finally, the treatment of nuisance parameters (here, the rate change parameters) is fundamentally different. While in the model-averaging approach, inferences are conditioned on Maximum-Likelihood point estimates of nuisance parameters, the Bayesian approach integrates out nuisance parameters, giving marginal probabilities of species limits. These final two differences are intrinsic to Bayesian inference, and researchers choosing among methods will need to consider their choice of statistical paradigm. We note that our confidence intervals in all analyses (including from the Likelihood method) are far wider than those of Pons et al., which were 46–51 species. This is most likely because of the difference in obtaining ultrametric trees. Pons et al. used non-parametric rate smoothing [55] on a Maximum Likelihood estimate of the tree. While they achieved sensible results with narrower confidence limits than ours, we nevertheless advocate an approach that samples trees and fits them to a clock model using a parametric method. This allows for a full accounting of uncertainty associated with phylogenetic estimation, albeit at the cost of some precision.

## Conclusions

Our results demonstrate that the Bayesian implementation of the GMYC model is reasonably reliable given two caveats. First, the length of the DNA sequence is important. We found that when we sampled only 300 bp, or only 2 alleles per species, that the performance of the model declined strongly. Second, the model is only useful when the underlying history of the species under consideration lies in particular regions of parameter space. Species that have recently diverged, or clades undergoing rapid radiation are unlikely to be identifiable under the model. In the latter case, the model may provide misleading estimates and confidence. Cases such as these, however, may be recognizable because the results may be highly unexpected in the context of other sources of data such as morphology or geography.

Our implementation of the model provides two main improvements over the original. First, it allows the specification of prior probabilities on model parameters. It is our experience that very high values of the Yule process rate change parameter sometimes have high likelihood and result in high uncertainty in the threshold parameter (unpublished empirical data). These high values may be biologically unrealistic, and the specification of an informative prior can reduce the posterior probability of those areas and produce a more accurate estimate of diversity. Second, it allows for the characterization of species limits without use of a point estimate of the phylogeny. We know that many datasets are associated with substantial uncertainty owing to limited sequence data collection. The Bayesian GMYC method provides marginal probabilities of species identities and will allow downstream estimates of species diversity and community structure (which are often the goal of environmental sequencing studies; [32]) to account for uncertainty underlying species designations.

An important future direction for this work is to implement the multiple-threshold version of the model proposed by Monaghan et al. [28], which can account for greater variation in divergence times and effective population sizes than the model implemented here. It has been shown to provide a better fit to some datasets [32], but will require implementation of a more complex reversible-jump MCMC that allows proposals that change the number of parameters in the model.

It is widely acknowledged that single-locus data are not optimal for the inference of phylogeny, historical demography, or species limits [56-59]. Nevertheless, vast amounts of biological diversity remain undescribed at the level of species, and this limits our ability to understand the evolutionary history of our planet and its current ecological functioning. Available alternative means of describing species diversity, either from molecular or morphological data have major drawbacks in that they are time consuming, expensive, or subject to their own biases and inaccuracies. Single-locus data for many groups are currently being generated on a large scale, and we advocate making the best of this data. We believe that under certain conditions, the GMYC model can be useful, and that a Bayesian framework accounting for uncertainty is most appropriate for these data.

## Competing interests

The authors declare that they have no competing interests.

## Authors’ contributions

NR and BC developed the original concept and designed the simulation experiments. NR wrote all software and conducted the analyses described in the manuscript. NR and BC interpreted results and wrote the manuscript. All authors read and approved the final manuscript.

## Supplementary Material

Additional file 1 **Figures S**1**, S**2**, S**3**.** These figures display the distribution of MCMC samples for each treatment and each replicate within treatments for simulated data. **S1** is results from the tree depth simulation, **S2** is the results from the allele sampling simulation and **S3** is the results from the nucleotide sampling simulation. Click here for file
