# Supplementary material for: Phylogenetic estimation error can decrease the accuracy of species delimitation: a Bayesian implementation of the general mixed Yule-coalescent model
Source: BMC Evol Biol. 2012 Oct 2;12:196. doi: 10.1186/1471-2148-12-196 (PMC3503838; doi:10.1186/1471-2148-12-196)
Supplement: Additional file 1 — Figures S1, S2, S3. These figures display the distribution of MCMC samples for each treatment and each replicate within treatments for simulated data. S1 is results from the tree depth simulation, S2 is the results from the allele sampling simulation and S3 is the results from the nucleotide sampling simulation. [file 1471-2148-12-196-S1.pdf]

S1

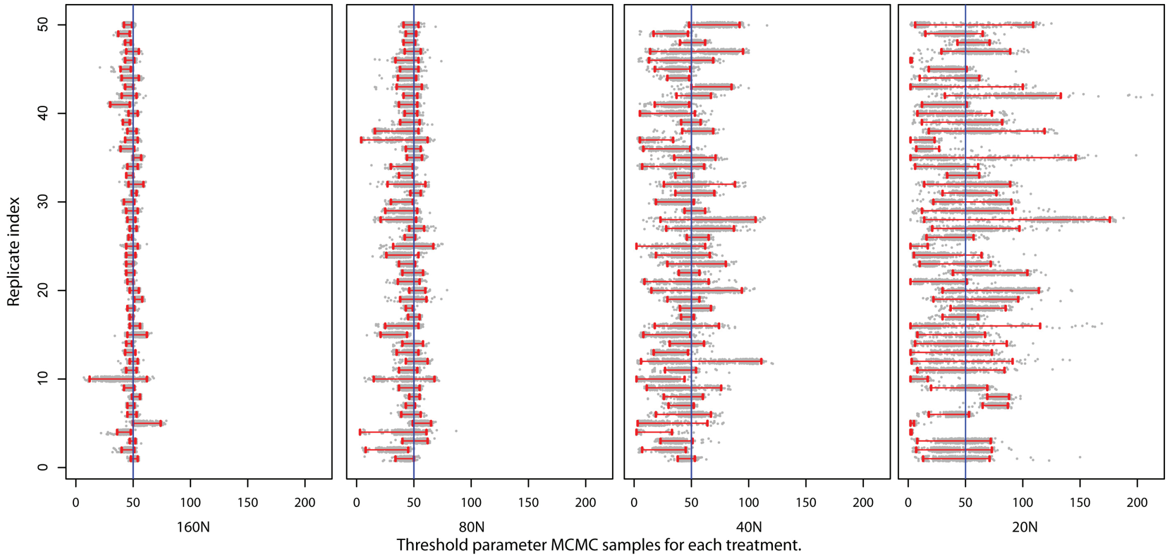


S2

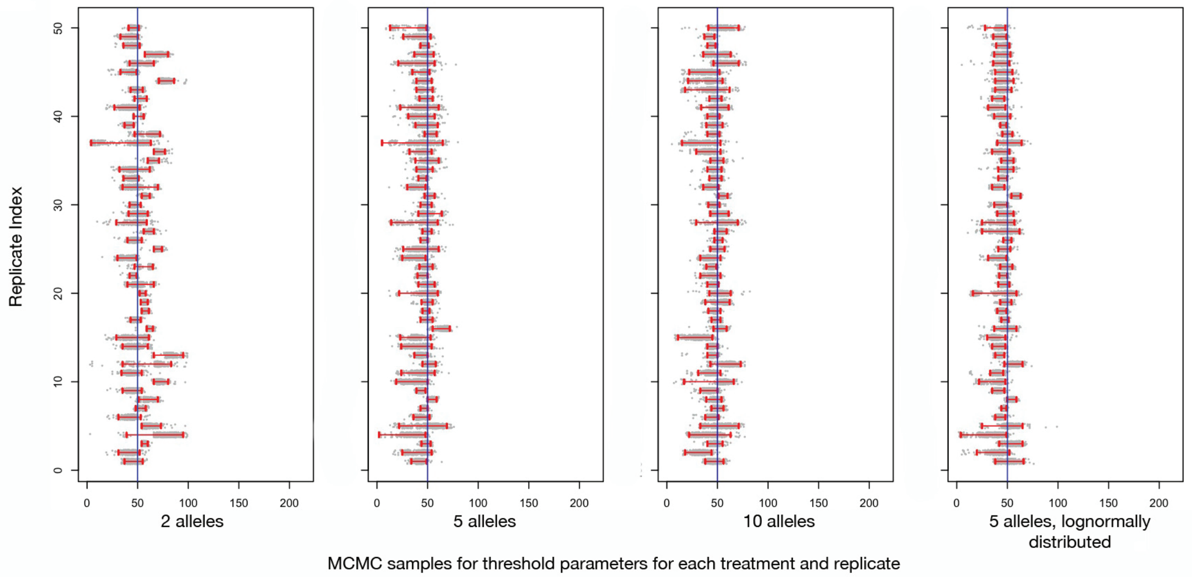


S3

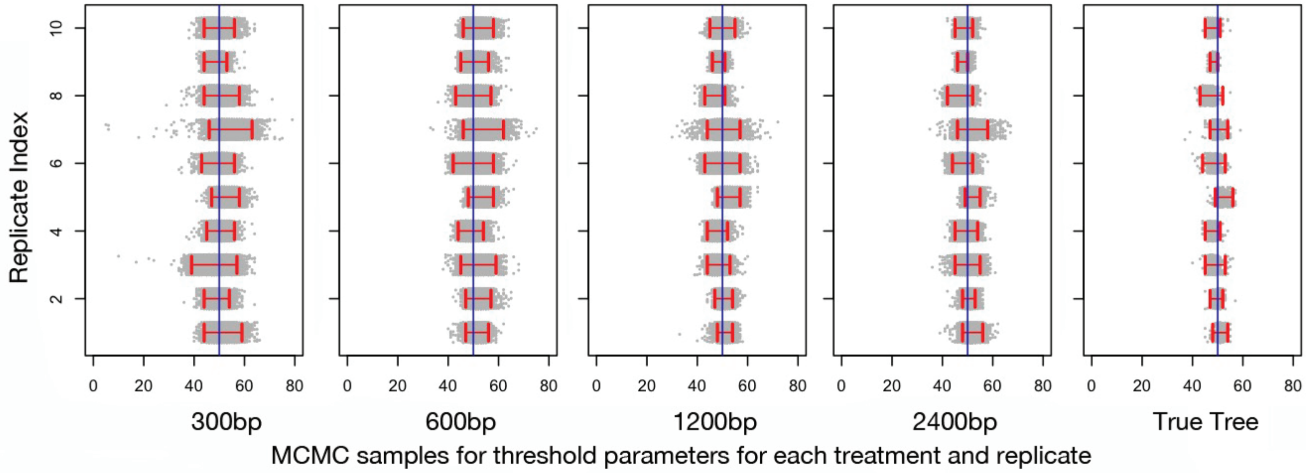


**Supplementary Figures**: These figures display the distribution of MCMC samples for each treatment and each replicate within treatments for simulated data. S1 is results from the tree depth simulation, S2 is the results from the allele sampling simulation and S3 is the results from the nucleotide sampling simulation.
